# An error-corrected linear approximate almost ideal demand system model for imported meats and seafood in Indonesia

**DOI:** 10.1016/j.heliyon.2023.e21390

**Published:** 2023-10-30

**Authors:** David Forgenie, Nikmatul Khoiriyah, Meera Mahase-Forgenie, Bosede Ngozi Adeleye

**Affiliations:** a Department of Agricultural Economics and Extension, Faculty of Food and Agriculture, The University of the West Indies, St. Augustine Campus, Trinidad; b Department of Agribusiness, Faculty of Agriculture, University of Islam Malang, Malang, Indonesia; c Department of Geography, Faculty of Food and Agriculture, The University of the West Indies, St. Augustine Campus, Trinidad; d Department of Accountancy, Finance and Economics, University of Lincoln, Lincoln, United Kingdom

**Keywords:** Elasticities, Error-correction, Imported meats, Indonesia, Seafood

## Abstract

The use of static-demand systems in empirical analysis assumes that consumers adjust immediately to a new long-run equilibrium path when a shock is encountered. However, adjustment is not always instantaneous. This paper utilizes an Error-Corrected Linear Approximate Almost Ideal Demand System (EC-LAAIDS) model to analyze the import demand for meat and seafood in Indonesia from 1976 to 2020 using annual data. The study found that the theoretical restrictions of homogeneity and symmetry held in the EC-LAAIDS model but did not in the long-run model. The adjustment parameter reveals that imported mutton had the slowest adjustment to long-run equilibrium, while all other meats and seafood had a moderate adjustment speed. The study calculated income elasticities for both the short- and long-run revealing that imported beef was a luxury good in the short-run and most responsive to changes in income. In the short-run, imported poultry was the least responsive to changes in income. In the long-run, all imported meats were found to be luxuries except for imported seafood and beef. Uncompensated price elasticity of demand reveals that in the short-run all imported items had inelastic demand except for imported beef which had elastic demand. In the long-run, however, imported beef and pork had elastic demand, while all other items were inelastic. Compensated cross-price elasticities found that mostly substitution relationships existed among pairs of imported commodities. Finally, a few policy suggestions were discussed, such as production subsidies to producers.

## 1 Introduction

In 2010 the global consumption of meat and seafood products was estimated to be around 209.20 million tonnes which grew to around 248.73 million tonnes in 2019 [1]. Global per capita consumption of meat and seafood products between 2010 and 2019 has increased by around 3.9% on average [2]. Consumption is expected to continue to increase as population and income grow annually [3]. Whitton et al. [4] also note that there is a direct relationship between development and increased meat consumption. According to OECD/FAO [3], the availability of protein from beef, pork, poultry and sheep meat is projected to increase by around 5.9%, 13.1%, 17.8%, and 15.7% by 2030, respectively. Current consumption patterns suggest that consumers are shifting meat consumption toward poultry, especially in developing nations with low-income levels [3]. Furthermore, it is projected that by 2030 poultry meat will account for around 41% of all protein derived from animal sources globally [3].

The global trade of meat and seafood products has also been impacted by several challenges, including outbreaks of animal diseases, trade restrictions due to food safety concerns, and fluctuations in exchange rates [[5], [6], [7]]. These challenges have led to increased uncertainty in the market and have made it more difficult for countries to manage their meat and seafood trade. Despite these challenges, the global trade of meat and seafood products has grown in recent years, driven by the increasing demand for these products in developed and developing countries [[8], [9], [10], [11]]. This growth is expected to continue as consumers worldwide continue to seek high-quality, safe and sustainable meat and seafood products.

Consumption of livestock products in developing countries, especially beef and poultry, is increasing due to population growth, urbanization, changing consumer preferences, and economic growth [[12], [13], [14], [15]]. However, most developing nations depend heavily on international trade to meet their demands. In Indonesia, current statistics show that domestic production of some meat groups needs to catch up to domestic consumption demands [[15], [16], [17]]. Meat consumption represents an essential source of high-quality dietary protein for most of the global population [18,19]. However, according to Von Grebmer et al. [20] and Khoiriyah et al. [21], Indonesia is designated as a nation with severe hunger and malnutrition, which can be attributed in part to inadequate protein consumption from animal sources. The national per capita daily recommended protein intake set by the Indonesian government is 57 grams [22,23]. However, low- and middle-income households still need to meet this daily recommended requirement in urban and rural areas which is below the national standard. Hence, there is a need for policy action to help improve animal-derived protein food and nutritional security for households in Indonesia.

Beef and poultry are two of the most widely consumed meat groups in Indonesia since most consumers are Muslims. Per capita, beef consumption in 2019 was approximately 2.16 kg per day [24]. However, domestic production cannot adequately meet consumer demand [15]. Between 2010 and 2020, beef consumption increased by approximately 43.7%, while domestic production only increased by 36.9% [24]. Therefore, Indonesia increased imports by around 56.7% to meet the domestic demand for beef [15]. Given this scenario, dependence on imports for beef to meet consumer demand is a serious problem and poses a severe threat to the food security of Indonesian households. Furthermore, the recent COVID-19 pandemic has caused much disruption to the global food network [[25], [26], [27]], which resulted in significant price increases for many imported items, which has severe implications for food security and welfare. For instance, the FAO food price index shows that between 2018 and 2020, the world price of imported meat increased by approximately 5.3% on average [28]. Furthermore, in recent times, domestic beef prices have been consistently higher than international prices [15]. This further widens the gap in Indonesia's minimum daily protein consumption.

Excessive importation of beef to satisfy domestic consumer demands creates much uncertainty for food and nutrition security in Indonesia because shocks to the global food system brought about by natural disasters, political instability, increased production cost, war, and climate change are transferred to Indonesian consumers in the long-run in the form of higher prices which negatively affects welfare. This is because Indonesia has a large Muslim population that depends heavily on beef consumption for protein [29,30]. In addition, the over-dependence on one country, Australia, for meat imports has become an issue of significant concern for Indonesian consumers [12]. Hence, efforts are made by the Indonesian government to improve meat production, mainly beef production. For instance, to set the direction of food and nutrition security policy, the government has defined beef as one of the seven strategic food commodities to improve production to meet domestic consumption demands [[31], [32], [33]]. However, this policy has yet to help curb the ever-increasing beef import bill. In order to ensure that the minimum per capita daily protein requirement is met, particularly those in low- and middle-income households, policies geared towards reducing excessive meat imports are needed.

Ever since the proposal of the almost ideal demand system (AIDS) model by Deaton and Muellbauer [34], a vast body of literature utilizing the demand system approach to study consumer behavior has developed, for example, Kharisma et al. [19], Taljaard et al. [35], Wadud [36], Alboghdady and Alashry [37], Udoh et al. [38], Sola [39], Zhou [40], Akinbode [41], Lecocq and Robin [42], Nankwenya et al. [43], Anindita et al. [44], Khoiriyah et al. [45], and Forgenie et al. [46]. Studies on meat and seafood demand estimation have been carried out in many countries; see Taljaard et al. [35], Chen and Veeman [47], Mdafri and Brorsen [48], Karagiannis et al. [49], Verbeke and Ward [50], Jabarin [51], Basarir [52], Motallebi and Pendell [53], Zhou [54], Shibia et al. [55], Anindita et al. [56], Selvanathan et al. [57], Khoiriyah et al. [58], Rianti and Khoiriyah [59], and Khoiriyah et al. [60] using the AIDS approach. However, most of these studies examine demand at the household level. Therefore, studies that focus on import demand estimation can be extremely valuable for the importing country and their trading partners as a comprehensive understanding of how factors such as prices and income affect demand can be better understood.

In addition, most studies using the AIDS model tend to ignore the time-series properties of the data and estimate the static AIDS model explicitly. However, according Shrestha and Bhatta [61], most economic time-series are nonstationary and overlooking this property can lead to spurious results. In addition, Chang and Nguyen [62] and notes that ignoring the dynamic properties of time-series data can lead to the theoretical restrictions of homogeneity and symmetric, which is usually imposed on the AIDS model being rejected. This has led Rathnayaka et al. [63], Singh et al. [64], and Nzuma and Sarker [65] to estimate an error-corrected version of the AIDS model to account for the dynamic properties of the data. However, no study has applied the error-corrected linear approximate AIDS model to study meat and seafood import demand in Indonesia.

Therefore, this paper presents an Error-Corrected Linear Approximate Almost Ideal Demand System (EC-LAAIDS) as used by Singh et al. [64] to study the import demand for meat and seafood in Indonesia over the period 1976–2020. The EC-LAAID model considers the dynamic nature of the Indonesian food market and includes an error-correction mechanism to capture the effects of short- and long-run deviations from the equilibrium relationship between demand and relevant variables (price and income). We also estimate short- and long-run price and income elasticities of demand for the various imported meats and seafood. The findings of this study have profound policy implications as they can be used by government and policymakers as it provides price and income elasticities for both the short- and long-run, which can be used as tools to effect policies specific to both time horizons. Additionally, our findings will contribute to the growing body of research on the economics of food demand and provide a foundation for future studies on the Indonesian food market.

The remainder of this paper is organized into materials and methods where the appropriate model and econometric techniques are explained, followed by the results and discussion section where we present and discuss the findings of our study. We then discuss the policy implications of our results, followed by a conclusion.

## 2 Materials and method

### 2.1 The linear approximate almost ideal demand system (LA-AIDS) model

Studies on demand analysis are widely published in the empirical literature. The demand for imported meat and seafood in Indonesia is estimated using a two-stage budgeting approach that assumes Indonesia's preferences are weakly separable with respect to other imported foods. In the first step, Indonesia decides how much of its total spending will go toward imported meat, seafood, and other consumer items. The prices of individual imported meats and seafood and total expenditure were used to determine the import demand for each imported commodity in the second stage. The demand for imported meat and seafood can then be estimated using a system of demand approach using the almost ideal demand system (AIDS) model developed by Deaton and Muellbauer [34] in the second stage of the two-stage budgeting procedure.

The AIDS model is based on a utility function that is defined as a second-order approximation of any utility function. Deaton and Muellbauer [34] begin by specifying an expenditure function that belongs to the PIGLOG class of preferences that meets the requirements for consistent aggregation across customers. These requirements ensure that the functional forms of the market demand equations correspond to the behavior of a rational representative economic agent [34]. Ever since Deaton and Muellbauer [34] proposed the AIDS model, researchers have favored the specification over other functional forms due to its many favorable properties. Barnett and Seck [66] highlight that the AIDS model aggregates perfectly over consumers, has a functional form consistent with available data, satisfies the axiom of choice, is relatively easy to estimate, and allows theoretical restrictions of homogeneity and symmetry to be imposed and tested empirically. Although many of the existing functional forms in the literature possess many of the desirable properties noted above, only the AIDS model possesses all of them at the same time [34,66,67]. This makes the AIDS model extremely popular among scholars studying consumer demand [68,69].

The AIDS model in budget share for imported meat and seafood commodities is specified as follows:

(1) $$S_{it}=\alpha_{i}+\beta_{i}\;\ln\left(\frac{M_{t}}{P_{t}}\right)+\sum_{j}^{n}\gamma_{ij}{lnp}_{jt}+\varepsilon_{it}$$

Where *S*_*it*_ is the budget share of the *i*th imported commodity derived by dividing the import expenditure of the *i*th commodity group by the total import expenditure of all imported commodities. *P*_*jt*_ is the import price of the *j*th imported commodity. *M*_*t*_ is the total import expenditure on all imported commodities, while epsilon ($\varepsilon_{it}$) is a white noise error term. ${\text{Therefore},\alpha }_{i}$, $\beta_{i}$ and $\gamma_{ij}$ are parameters to be estimated. In addition, *P*_*t*_ is an aggregate price index which is defined in the traditional AIDS model as:

(2) $$\ln\;P_{t}=\alpha_{0}+\sum_{i=1}\alpha_{i}{lnp}_{it}+\frac{1}{2}\sum_{i=1}\sum_{j=1}\gamma_{ij}\;\ln\;p_{it}{lnp}_{jt}$$

In order to ensure that the AIDS model is consistent with economic theory, three parametric restrictions are imposed upon the parameters as suggested outlined by Deaton and Muellbauer [34], namely adding-up, homogeneity, and symmetry highlighted in equations (3), (4), (5):

(3) $$\text{Adding}-\text{Up}:\sum_{i=1}^{n}\alpha_{i}=1;\;\sum_{i=1}^{n}\beta_{i}=0;\;\sum_{i=1}^{n}\gamma_{\text{ij}}=0$$

(4) $$\text{Homogeneity}:\sum_{j=1}^{n}\gamma_{\text{ij}}=0$$

(5) $$\text{Symmetry}:\gamma_{ij}=\gamma_{ji}\;for\;i,j=1,2,3,4,5\;and\;i\neq j$$

The adding-up restriction is automatically imposed since the budget shares must sum to unity; hence, one share equation is omitted during estimation, and the parameters of the omitted share equation are recovered using the adding-up restriction. Homogeneity and symmetry restrictions are imposed during estimation.

The price index specified in equation (2) raises some empirical issues, primarily when aggregate annual time-series data is utilized. According to Taljaard et al. [35] and Rathnayaka et al. [63], the use of the price index specified in equation (2) makes the demand system non-linear, which complicates the estimation process. Hence, to overcome the non-linearity problem, Deaton and Muellbauer [34] ascertain that the stone geometric price index can be used to linearize the model, which is specified as:

(6) $$\ln\;P_{t}=\sum_{i}S_{it}{lnp}_{it}$$

Substituting the price index in equation (6) into equation (1) yields the linear approximate AIDS (LA-AIDS) model. The LA-AIDS model has been widely applied in demand analysis, for example, Alboghdady and Alashry [37], Zhou [40], Anindita et al. [44], Verbeke and Ward [50], Jabarin [51], Shibia et al. [55], Basarir [70], Khan [71], Rahman et al. [72], Siddique et al. [73], Mufeeth et al. [74], Agwaya and Ochieng [75], da Silva Pinto et al. [76], Gido [77]. The LA-AIDS model for imported meats and seafood is estimated using iterated seemingly unrelated regression (ISUR) by Zellner [78] to account for cross-equation correlation. In addition, to avoid singularity of the variance co-variance matrix, one of the share equations is omitted during estimation and recovered post-estimation using adding-up restriction.

### 2.2 Stationarity

One of the critical assumptions of the classical linear regression model (CLRM) is that the time series in question are stationary or do not have a unit root. According to Gujarati [79] and Kennedy [80], a time series is stationary with its stochastic properties between observations are consistent over time or time-invariant. However, Shrestha and Bhatta [61] note that most macroeconomic time series data tend to be nonstationary since they display strong upward or downward movement over time without any indication of reverting to a fixed mean. Stationarity of time-series variables is vital in empirical work since routine estimations using ordinary least squares (OLS) estimation can result in spurious regression [81]. The use of spurious results to inform policy decisions can ultimately lead to ineffective policy recommendations. Hence, it is crucial that time series are evaluated for unit roots before they are used in empirical estimations. Test for unit root can be carried out using the augmented Dickey-Fuller (ADF) test [82,83], Phillips and Perron (PP) [84], and the KPSS test [85]. However, the ADF is one of the most popular unit root tests used in the empirical literature [86,87]. The test can be performed with and without a trend on the level variables. If it is discovered that the series in question has a unit root, then it is differenced *d* number of times to make it stationary. This article utilized the ADF test with and without trends to ascertain if the series are all generated by a stationary process.

### 2.3 Cointegration analysis and error corrected LA-AIDS

To negate the negative implications of spurious regression by using nonstationary time series variables, nonstationary variables are differenced to make them stationary. However, differencing can lead to valuable long-run relationships between variables being lost that would have been otherwise given by the level variables [79]. Shrestha and Bhatta [61] ascertain that two or more variables may form a long-run equilibrium relationship despite deviations from equilibrium in the short-run. Furthermore, Enders [88] notes that if a group of times series data has an equilibrium relationship, they cannot move independently from each other; hence, they are cointegrated. Cointegration analysis enables us to determine the long-run equilibrium relationship among nonstationary variables. This is because any short-run deviation will die out gradually, and long-run equilibrium will be achieved.

Cointegration test can be conducted using the Engle and Granger [89] two-step approach. In this approach, the long-run model is estimated, then the residuals are obtained, and the ADF test is used to test for cointegration. If the ADF test reveals that the estimated residuals are stationary, then the variables in question are cointegrated and share a long-run equilibrium relationship, and the results are not spurious. Banerjee et al. [90] also propose a dynamic test for cointegration where the error-correction term (ECT) in the error-correction model (ECM) is evaluated for statistical significance using the standard *t*-test. If the t-statistic for the ECT is statistically significant, then there is evidence that the variables are cointegrated. This study utilizes both the ADF and dynamic test for cointegration. If the variables are cointegrated, then an error-corrected LA-AIDS (EC-LAAIDS) model is specified to separate the long- and short-run dynamic of imported meat and seafood demand in Indonesia. Following Singh et al. [64], the EC-LAAIDS model for Indonesian meat and seafood demand is as follows:

(7) $${\Delta S}_{it}=\alpha_{i}+\beta_{i}\Delta \ln\left(\frac{M_{t}}{P_{t}}\right)+\sum_{j}^{n}\gamma_{ij}{\Delta lnp}_{jt}+\zeta_{i}\Delta S_{it-1}+\lambda_{i}{ECT}_{t-1}+\varepsilon_{it}$$

In equation (7), Δ is the difference operator, $S_{it-1}$ is a one-period lagged dependent variable included to incorporate habit effects in the model, and ${ECT}_{t-1}$ is the lagged residuals of equation (1) lagged one period. Lambda ($\lambda_{i})$ is the error-correction term that measures the speed of adjustment of short- to long-run equilibrium. It measures how much of the short-run equilibrium is corrected each period and shows how long the estimated parameters take to return to their long-run equilibrium state. Equation (7) is estimated with the theoretical restrictions of homogeneity and symmetry imposed using iterated seemingly unrelated regression estimation (ISURE) by Zellner [78].

The estimated parameters in equations (1), (7) can be used to derive import income and price elasticities of demand for both the long- and short-run, respectively. Using formulas by Chalfant [91], the income elasticity of demand for the various imported meats and seafood by Indonesia is calculated as follows:

(8) $$\eta_{i}=\frac{\beta_{i}}{s_{it}}+1,i=1,\ldots ,n..$$

Own- and cross-price elasticities are derived using the formulas outlined in equations (9) and (10) as follows:

(9) $$\varepsilon_{ij}^{M}=\frac{\gamma_{i}}{s_{it}}-\frac{\beta_{i}s_{jt}}{s_{it}}-\delta_{ij},i,j=1,\ldots ,n\text{.}$$

(10) $$\varepsilon_{ij}^{H}=\varepsilon_{ij}^{M}+\eta_{i}s_{it}$$

The formulas outlined in equations (8), (10) is provided by Where $\eta_{i}$, $\varepsilon_{ij}^{M}$ and $\varepsilon_{ij}^{H}$ are the income, uncompensated, and compensated elasticity of import demand, respectively. $s_{it}$ is the mean budget share of the *j*th import commodity, and $\delta_{ij}$ is the Kroncher delta which takes the value of 1 for own-price elasticity and 0 otherwise.

### 2.4 The data and source

This study utilized annual import quantities and expenditure data for the 1976–2020 (*T*=*44*) for Indonesia. All quantities are in metric tonnes, and expenditures and prices are in US dollars. The study included five aggregated commodity groups which included imported seafood (fish, shrimp, lobster, and other shell-fish), beef (fresh and frozen), pork (fresh and frozen pig meat), poultry (chicken, turkey, eggs, and ducks), and mutton (goat and lamb both fresh and frozen). Import prices were unavailable for imported meats and seafood categories; hence, import unit value was utilized by dividing total expenditure on the commodity group by total expenditure on all imported commodities. Data for imported meats (beef, pork, poultry, and mutton) was obtained from the Food and Agriculture Organization of the United Nations online database. Import data for seafood was obtained from the FISHSTAT J database. All empirical estimation was done using STATA 17.

**Fig. 1:**
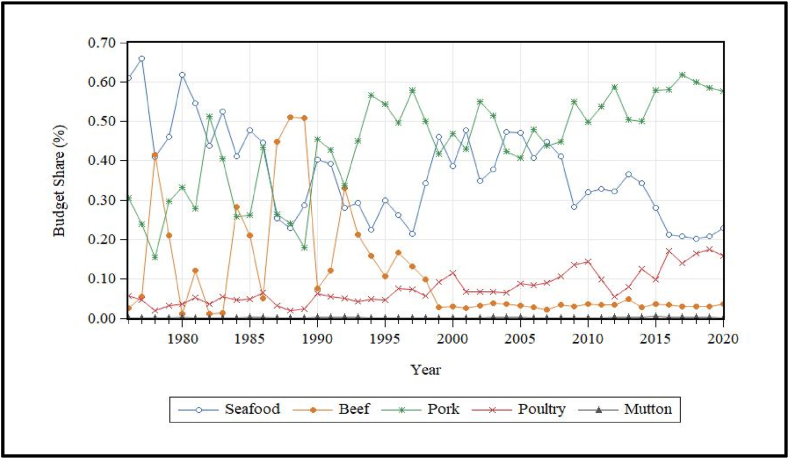
Import share of imported meat and seafood in Indonesia, 1976–2020.

Table 1 presents the descriptive statistics for the five imported commodity groups. Table 1 reveals that imported pork had the highest average annual import share for the study period. Between 1976 and 2020, the average import expenditure on imported beef was around 44%, followed by imported seafood, around 36.9%. On the other hand, imported mutton had the least average expenditure for the study period, which was less than 1% on average. Generally, for the study period, imported seafood and pork had the highest export expenditure share while imported mutton products had the lowest (Fig. 1.). Trends in import expenditure for seafood seem to be decreasing in recent years decreasing from 36.5% in 2013 to around 22.8% in 2020 (Fig. 1.). Imported mutton on the other hand has seen vary little variation in import share expenditure over the study period compared to imported beef which accounted for around 16.7% of total meat and seafood import expenditure in 1996 to only around 0.04% in 2020 (Fig. 1).

**Table 1:** Summary statistics for imported meats and seafood.

Variable	Mean	Std. Dev.	Minimum	Maximum
Budget Share				

S_1_	0.369	0.116	0.202	0.659
S_2_	0.111	0.135	0.010	0.510
S_3_	0.440	0.123	0.154	0.617
S_4_	0.077	0.041	0.019	0.175
S_5_	0.002	0.000	0.000	0.005

Unit Price (US$ per Metric Tonne)				

p_1_	1925.96	752.57	907.39	3819.53
p_2_	868.01	462.89	338.13	2203.53
p_3_	599.89	259.09	175.24	1266.62
p_4_	2313.87	1098.91	955.01	4611.15
p_5_	3322.39	1800.04	1106.42	7059.72

Note: S=budget share and p=price, 1=seafood, 2=beef, 3=pork, 4=poultry, and 5=mutton.

**Fig. 2:**
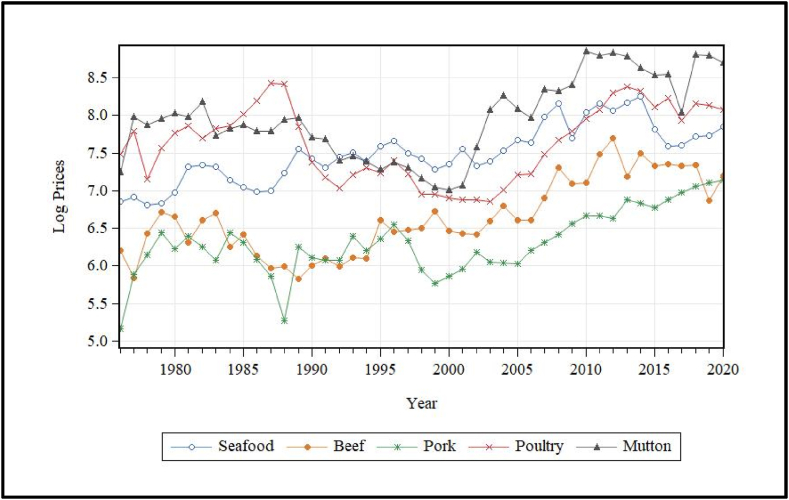
Log import price of imported meat and seafood, 1976–2020.

In terms of import price, imported mutton was found to be the most expensive per metric tonne on average, followed by imported poultry (Table 1). On average, imported mutton was almost twice as expensive per metric tonne than imported seafood, beef and poultry. However, on average, imported mutter was five times more expensive per metric tonne than imported beef and pork. On average, imported pork was the least expensive imported meat group by Indonesian consumers for the study period. Fig. 2. shows that the import price of pork is increasing over time relative to the import price of other imported meats and seafood. Imported poultry products have seen major surges in import prices between 2004 and 2020, increasing from around US$426.51 per metric tonne in 2004 to around US$1266.62 in 2020 (Fig. 2.). Generally, all imported commodities have seen major increase in import prices from 2000 onwards.

## 3 Results and discussion

### 3.1 Stationarity and cointegration test

As stated prior, it is paramount that the data's time-series properties be investigated to ensure that the results are meaningful. The ADF, PP, and KPSS tests were used to determine if the data was nonstationary. However, since the three tests yielded similar results only the results of the ADF test is presented in Table 2 and the results of the PP and KPSS tests are presented in Table A1 and A2 in Appendix A. The test was performed with and without a trend term. For most of the level series, the null hypothesis, which states that the level variable contains a unit root, is not rejected at the conventional 5% significance level. However, when the ADF test was performed on the variables' differences, the unit root null hypothesis was rejected at the 5% significance level. The ADF test suggests that most variables are nonstationary in level form; however, they are stationary in the first difference or integrated of order one.

**Table 2:** Augmented dicky-fuller test for unit roots.

Series	Level Series		First Difference Series	
	No Trend	Trend	No Trend	Trend
Budget Shares				

S_1_	−3.069	−3.397	−7.627	−7.502
S_2_	−3.349	−4.574	−9.057	−8.902
S_3_	−2.332	−4.314	−8.552	−8.480
S_4_	−1.043	−3.709	−6.191	−6.153
S_5_	−3.530	−3.977	−5.950	−5.902

Log Price and Expenditure				

lnp_1_	−1.919	3.270	−6.171	−6.189
lnp_2_	−1.529	−2.087	−6.630	−6.537
lnp_3_	−1.529	−2.384	−5.632	−5.609
lnp_4_	−1.486	−1.615	−3.889	−3.765
lnp_5_	−0.942	−1.653	−4.776	−4.816
ln (M/P)	−1.391	−5.083	−7.391	−7.294

Critical Values				

1%	−3.628	−4.214	−3.634	−4.224
5%	−2.950	−3.528	−2.952	−3.532
10%	−2.608	−3.197	−2.610	−3.199

Note: ln=natural logarithm, S=budget share and p=price, 1=seafood, 2=beef, 3=pork, 4=poultry, and 5=mutton.

Since the ADF test found that the variables are integrated of order one, it is paramount that cointegration is investigated to ascertain a long-run equilibrium relationship among the variables. To determine if the budget shares are jointly determined by their respective prices and income, cointegration test was conducted using one residual-based test (ADF test) and a dynamic cointegration test. The results of both tests for cointegration are presented in Table 3. The results of the ADF test show that the hypothesis of no cointegration was rejected at the 10% significance level for the budget shares of all imported meat and seafood commodities. Furthermore, the results of the dynamic test for cointegration also reject the hypothesis of no cointegration, as all of the t-values associated with the error-correction term (ECT) from the conditional error-correction models (ECM) are all statistically significant. Therefore, there is enough evidence to suggest that there is a long-run equilibrium relationship among the variables. Therefore, cointegration indicates that the variables all move together in the long-run and obey an equilibrium constraint [61,63,64].

**Table 3:** ADF and dynamic test for cointegration.

Share Equation	ADF Cointegration Test		Dynamic Cointegration Test	
	No Trend	Trend	EC Term	t-value
Seafood	−4.709	−4.949	−0.532	−7.650
Beef	−6.065	−6.417	−0.534	−7.650
Pork	−4.685	−4.785	−0.533	−7.630
Poultry	−5.107	−5.322	−0.525	−7.450
Mutton	−4.896	−4.902	−0.401	−6.080

Critical Values				

1%	−5.281	−5.585	–	−2.692
5%	−4.710	−5.028	–	−2.015
10%	−4.431	−4.731	–	−1.680

NB: Critical values for ADF and PP Cointegration test obtained from Phillips and Ouliaris (1990).

### 3.2 Theoretical restriction test and model parameters

The long-run and error-corrected version of the LA-AIDS model is estimated using equations (1), (7), respectively. The models were estimated with the theoretical restrictions of homogeneity and symmetry imposed using iterated seemingly unrelated regression (ISUR) procedures. Since the budget shares in equations (1), (7) should sum to unity, one of the share equations is omitted during estimation to prevent singularity of the residual's variance-covariance matrix. The parameters of the omitted share equation are then recovered using the adding-up restriction.

One advantageous property of the AIDS model is that it allows for the theoretical restrictions of homogeneity and symmetry to be imposed and tested empirically. This study used the likelihood ratio (LR) test to test the hypothesis of homogeneity and symmetry in the system of import demand equations given in equations (1), (7) and the significance of the restricted model versus the unrestricted model. The results of the LR test for both the long-run and EC-LAAIDS models are presented in Table 4. The computed chi-squared value from the LR test for the long-run and EC-LAAIDS models were 32.16 and 17.32, respectively. Compared to the critical chi-squared value at the 5% significance level, which was 18.31, it can be concluded that the linear homogeneity and symmetry hypothesis did not hold in the long-run LAAIDS model but held in the EC-LAAIDS model. The results suggest that the empirical obtained by the EC-LAAIDS model are theoretically consistent and valid for this specification. However, according to Chang and Nguyen [62], Chang [92], rejection of the theoretical restrictions in the long-run LAAIDS model does not mean that the underlying theory is incorrect but can be due to ignoring the time-series properties of the data. Furthermore, it is typical for the theoretical restrictions of homogeneity and symmetry to not hold in the long-run model due to ignoring the time-series properties of the data [62]. Cozzarin and Gilmour [93] found that more than half of the studies that utilized the LA-AIDS model had the theoretical restriction rejected.

**Table 4:** Likelihood ratio test for parametric restrictions.

Parametric Restriction	Degrees of Freedom	Chi-Square Value	Critical Value	
			5%	10%
Long-Run LA-AIDS				

H_0_: Homogeneity	4	9.83	9.488	7.779
H_0_: Symmetry	6	13.96	12.592	10.645
H_0_: Homogeneity and Symmetry	10	32.16	18.307	15.987

Error-Corrected LA-AIDS				

H_0_: Homogeneity	4	8.07	9.488	7.779
H_0_: Symmetry	6	11.05	12.592	10.645
H_0_: Homogeneity and Symmetry	10	17.32	18.307	15.987

Note: 5% significance level is used to reject H_0_.

Table 5 presents the theoretically constrained results for the long-run and EC-LAAIDS models. It was found that most of the estimated parameters are statistically significant. The own-price of all imported meats and seafood was found to positively affect the import share equation in both time horizons except for beef, where it was found that own-price negatively affected the budget share. In the long-run model, the estimated income coefficients (*β*) for imported seafood and beef were negative, which suggests that these two imported items are necessities for Indonesian consumers. In contrast, the coefficients for imported pork, poultry and mutton were positive, suggesting that these imported items were luxuries in the long run. The short-run income parameters, however, suggested that all imported meats and seafood are necessities except for beef which was luxurious in the short-run.

**Table 5:** Estimated parameters of the long-run and EC-LAAIDS models.

Parameter	**Long-Run Import Share Equation**				
	**Seafood**	**Beef**	**Pork**	**Poultry**	**Mutton**
α	1.694 (0.141)	0.318 (0.238)	−0.688 (0.174)	−0.319 (0.059)	−0.004 (0.003)
**γ**_**1**_	0.014 (0.014)	0.143 (0.031)	−0.108 (0.026)	−0.048 (0.010)	−0.001 (0.000)
γ_2_	0.143 (0.031)	−0.222 (0.056)	0.058 (0.038)	0.022 (0.012)	−0.000 (0.000)
γ_3_	−0.106 (0.026)	0.058 (0.038)	0.035 (0.038)	0.014 (0.011)	0.000 (0.000)
γ_4_	−0.048 (0.010)	0.022 (0.012)	0.014 (0.011)	0.012 (0.007)	0.000 (0.000)
γ_5_	−0.001 (0.000)	−0.000 (0.000)	0.000 (0.000)	0.000 (0.000)	0.000 (0.000)
β_i_	−0.098 (0.010)	−0.025 (0.017)	0.091 (0.013)	0.032 (0.004)	0.000 (0.000)
R^2^	0.681	0.304	0.544	0.634	–
Chi-squared	111.62	33.73	79.09	95.68	–
Prob > Chi^2^	0.000	0.000	0.000	0.000	–
**Parameter**	**Error-Corrected Import Share Equation**				

**Seafood**	**Beef**	**Pork**	**Poultry**	**Mutton**
α	0.003 (0.007)	−0.013 (0.013)	0.008 (0.011)	0.002 (0.003)	1.000 (0.232)
γ_1_	0.105 (0.031)	0.046 (0.023)	−0.128 (0.023)	−0.022 (0.014)	−0.001 (0.001)
γ_2_	0.046 (0.023)	−0.159 (0.040)	0.117 (0.032)	−0.004 (0.011)	0.000 (0.000)
γ_3_	−0.128 (0.023)	0.117 (0.032)	0.001 (0.034)	0.011 (0.011)	−0.000 (0.000)
γ_4_	−0.022 (0.014)	−0.004 (0.011)	0.011 (0.011)	0.015 (0.011)	0.000 (0.000)
γ_5_	−0.001 (0.001)	0.000 (0.000)	−0.000 (0.000)	0.000 (0.000)	0.001 (0.003)
β_i_	−0.165 (0.020)	0.192 (0.040)	−0.024 (0.034)	−0.002 (0.009)	−0.000 (0.000)
ζ_i_	0.109 (0.060)	0.111 (0.060)	0.112 (0.061)	0.099 (0.061)	−0.429 (0.245)
λ_i_	−0.532 (0.070)	−0.534 (0.069)	−0.533 (0.069)	−0.525 (0.070)	−0.401 (0.066)
R^2^	0.759	0.620	0.416	0.388	–
Chi-squared	190.24	212.22	109.51	67.53	–
Prob > Chi^2^	0.000	0.000	0.000	0.000	–

Note: Standard errors are in parentheses.

In the EC-LAAIDS or short-run model, the parameter zeta (*ζ*) is included to account for the role of habit in Indonesian consumers' decision-making process. The results suggest that habit persistence plays a statistically significant role in determining Indonesian consumers' import demand for imported seafood, pork, and poultry in the short-run. Table 5 also presents the coefficients of the error-correction terms (ECT) given by lambda (*λ*) for each import expenditure share equation. It measures the speed of adjustment to recover the long-run equilibrium from a dynamic shock; the greater the magnitude of *λ* in absolute terms, the higher the speed of adjustment back to long-run equilibrium [64]. All the estimated ECTs are statistically significant and negative, as expected, indicating there is convergence to long-run equilibrium. The speed of adjustment parameters show that the short-run disequilibrium in import expenditure share for imported seafood, beef, pork, poultry, and mutton is corrected by around 53.2%, 53.4%, 53.3%, and 52.3%, and 40.1% on average annually towards long-run equilibrium, respectively. The number of periods (m) for a short-run shock to work its way through the market and attain long-run equilibrium can be predicted by m=(1– *λ*)/*λ* [64]. Hence, for imported seafood, beef, pork, and poultry, it takes around eight months on average for disequilibrium in import expenditure share to return to its long-run equilibrium state. However, imported mutton has a longer adjustment time towards long-run equilibrium, which was around one year and five months on average.

### 3.3 Income and uncompensated price elasticities

Income is one of the most critical determinants of import demand. Classical economic theory postulates that there is a direct relationship between income and demand [94]. As income increases, it is expected that consumption of goods and services will increase, and vice-versa. In order to measure the degree of responsiveness of demand brought about by changes in income, economists use income elasticity of demand. Income elasticities are interpreted with the ceteris paribus assumption imposed. The magnitude and sign of the income elasticity parameters give vital information regarding the nature of the good in question. For instance, if the estimated income elasticity of demand is positive, then the good is said to be normal, which means that an increase in income fosters an increase in demand. In contrast, the commodity is considered inferior if the estimated income elasticity is negative. For inferior goods, an increase in income leads to a decrease in demand. Table 6 presents income elasticities for imported seafood, beef, pork, poultry, and mutton for Indonesia in both the short- and long-run. Income elasticities were calculated using the estimated parameters from equations (1), (7) using the formula in equation (8). All of the estimated income elasticities were statistically significant and had a positive sign, meaning that all imported meats and seafood can be regarded as normal goods. However, most of the short-run income elasticities are smaller than their long-run counterparts except for imported beef which was larger in magnitude in the short-run. This is because, in the short-run, consumers tend to have less time to adjust to changes in prices or income; hence, elasticity parameters tend to be smaller [63,64].

**Table 6:** Income and uncompensated own-price elasticities.

Commodity	Income Elasticities		Own-price Elasticities	
	Short-Run	Long-Run	Short-Run	Long-Run
**Seafood**	0.553 (0.055)	0.734 (0.027)	−0.552 (0.087)	−0.865 (0.084)
**Beef**	2.731 (0.360)	0.778 (0.155)	−2.623 (0.372)	−2.978 (0.515)
**Pork**	0.945 (0.078)	1.206 (0.029)	−0.974 (0.078)	−1.010 (0.084)
**Poultry**	0.098 (0.127)	1.415 (0.053)	−0.804 (0.149)	−0.879 (0.095)
**Mutton**	0.702 (0.076_	1.229 (0.069)	−0.656 (0.163)	−0.896 (0.426)

Note: Standard errors are in parentheses.

In the short-run, imported beef was the most responsive to changes in income as the estimated income elasticity was 2.73, which is interpreted as a 1% increase in income of Indonesian consumers is expected to result in a 2.73% increase in the demand for imported beef. However, in the long-run, imported beef had an income elasticity of 0.78, which means that a 1% increase in income only results in a 0.78% increase in the demand for imported beef. Therefore, imported beef can be considered a luxury item in the short-run but not in the long-run. In the short- and long-run, imported seafood was found to be a normal good as a 1% increase in income results in a 0.55% and 0.73% increase in import demand, respectively. On the other hand, imported pork, poultry, and mutton were all found to be normal goods in the short-run, with income elasticities of 0.95, 0.10, and 0.70, respectively. Therefore, it was discovered that a 1% increase in income brings about a 0.95%, 0.10%, and 0.70% increase in the demand for imported pork, poultry and mutton in the short-run, respectively. However, in the long-run, imported pork, poultry, and mutton were found to be luxury items, as a 1% increase in income is expected to bring about a 1.21%, 1.42%, and 1.23% increase in the demand for these items, respectively. In the short-run, imported poultry was the least responsive to changes in income, while imported beef was the most responsive. In contrast, in the long-run, imported seafood was the least responsive to changes in income, while imported poultry was the most responsive.

This study also calculates uncompensated own-price elasticities of import demand for imported meats and seafood for Indonesia. Own-price elasticity of demand measures the degree of responsiveness of the quantity demanded brought about by changes in the price of the commodity. Economic theory dictates that there is an inversed relationship between the quantity of a commodity demanded and the prices; hence, it is expected that the calculated own-price elasticity of demand should be negative, although interpretation is made using the absolute form. The magnitude of the own-price elasticity also provides vital information regarding the behavior of consumers. For instance, own-price elasticities that are greater than unity have elastic demand, which means that the quantity demanded is highly responsive to price changes. In contrast, commodities with own-price elasticities that are less than unity have inelastic demand, meaning that the quantity demanded changes less than proportionate to price changes.

Uncompensated own-prices import elasticities of demand are presented in Table 6 for both time horizons. All calculated own-price elasticities for imported meats and seafood were found to be statistically significant and negative, as expected. In addition, all short-run own-price elasticities are smaller than their long-run counterparts. However, in both time horizons, imported beef was found to be the most responsive to changes in prices, as a 1% increase in prices is expected to bring about a 2.62% and 2.98% decrease in quantity demand in both the short- and long-run, respectively. Therefore, it was found that import demand for beef is price elastic for Indonesians in both time horizons. In contrast, imported seafood was found to be the least responsive to price changes in relation to other imported meat categories in both time horizons. For imported seafood, it was found that a 1% increase in prices is expected to bring about a 0.55% and 0.87% decrease in import quantity in the short- and long-run, respectively. Own-price elasticities for imported pork suggest that in the short-run, demand is price inelastic but is price elastic in the long-run. This is because a 1% increase in imported pork prices in the short-run brings about a 0.97% decrease in quantity demanded, while in the long-run, quantity demanded is expected to decrease by 1.01%. Imported poultry and mutton were found to be price inelastic in both time horizons.

### 3.4 Compensated price elasticities

The study also calculated compensated price elasticities of demand for imported meats and seafood in Indonesia. Compensated elasticities measure the responsiveness of quantity demand due to changes in the price of the commodity explicitly. Compensated or Hicksian elasticities provide a better measure of substitutability among commodities because they only consider the substitution effect [64]. The cross-price elasticity of demand measures the relationship between the quantity of a good demanded with the change in prices of another related good. A positive cross-price elasticity suggests that the relationship between the two commodities is substitution. This is because an increase in the price of one of the two related goods is expected to increase the demand for the other goods. However, if the cross-price elasticity of demand between the two related commodities is negative, then it is suggested that they are complements. Complementary goods have an inversed relationship when one of the prices changes. An increase in the price of one of the related goods leads to a decrease in demand for the other goods as they are usually consumed together. Table 7 presents compensated own-price elasticities for imported meats and seafood for both the short- and long-run. In both time horizons, all compensated own-prices elasticities are found to be negative and statistically significant, as expected. Compensated own-price elasticities for imported meats and seafood suggests that all imported commodities are price inelastic except for imported beef in both time horizons.

**Table 7:** Compensated import price elasticities.

Commodity	**Short-Run**				
	**Seafood**	**Beef**	**Pork**	**Poultry**	**Mutton**
**Seafood**	**−0.347 (0.084)**	0.235 (0.031)	0.094 (0.062)	0.019 (0.037)	−0.001 (0.001)
**Beef**	0.785 (0.204)	**−2.321 (0.365)**	1.494 (0.285)	0.037 (0.096)	0.005 (0.003)
**Pork**	0.079 (0.052)	0.376 (0.072)	**−0.558 (0.077)**	0.101 (0.024)	0.002 (0.001)
**Poultry**	0.090 (0.177)	0.054 (0.137)	0.578 (0.137)	**−0.728 (0.148)**	0.006 (0.005)
**Mutton**	−0.231 (0.187)	0.281 (0.164)	0.348 (0.156)	0.256 (0.193)	**−0.654 (0.157)**

**Commodity**	**Long-Run**				

**Seafood**	**Beef**	**Pork**	**Poultry**	**Mutton**

**Seafood**	**−0.593 (0.083)**	0.496 (0.085)	0.150 (0.070)	−0.053 (0.027)	0.000 (0.001)
**Beef**	1.658 (0.283)	**−2.891 (0.509)**	0.960 (0.342)	0.274 (0.112)	−0.001 (0.004)
**Pork**	0.126 (0.059)	0.242 (0.086)	**−0.479 (0.086)**	0.109 (0.024)	0.003 (0.001)
**Poultry**	−0.253 (0.129)	0.393 (0.160)	0.622 (0.139)	**−0.770 (0.096)**	0.007 (0.004)
**Mutton**	0.007 (0.006)	−0.066 (0.054)	0.659 (0.189)	0.293 (0.183)	**−0.894 (0.415)**

Note: Standard errors are in parentheses.

Cross-price elasticities for imported meats and seafood are also presented in Table 7. Most of the calculated cross-price elasticities are positive in both time horizons, which suggests that substitution relationships mainly exist among pairs of imported commodities. For instance, in the short-run, imported seafood was found to be substituted with imported beef, pork, and poultry when prices increased. A 1% increase in the price of imported seafood is expected to bring about a 0.24%, 0.09%, and 0.02% increase in demand for imported beef, pork, and poultry in the short-run, respectively. Imported beef benefits more from increased prices of imported seafood in the short-run. However, in the long-run, cross-price elasticities suggest that imported seafood and poultry are complements. A 1% increase in the price of imported seafood is expected to bring about a 0.05% decrease in demand for imported poultry. Imported beef was found to have a strong substitution relation with imported seafood and pork in both time horizons. In the short-run, a 1% increase in the price of imported beef is expected to bring about a 0.79% and 1.49% increase in the demand for imported seafood and pork, respectively. However, in the long-run, a 1% increase in the price of imported beef brings about a 1.66% and 0.96% increase in the demand for imported seafood and pork, respectively.

Imported pork mostly had weak substitution relationships with other imported meats and seafood, except for imported beef in both the short- and long-run. However, Indonesian consumers are more likely to increase consumption of imported beef in the short-run when imported pork prices increase. Imported poultry was found to have weak substitutability with imported seafood, beef, and mutton in the short-run but had moderate substitutability with imported pork. However, the substitutability of imported poultry for imported beef and pork was more significant in the long run. For instance, in the long-run, a 1% increase in the price of imported poultry is expected to bring about a 0.39% and 0.62% increase in the demand for imported beef and pork, respectively. In the long-run, however, imported poultry had a complementary relationship with imported seafood.

### 3.5 Policy implications

For policy impact on Indonesia, the role of government subsidy and taxation cannot be over-emphasized. Both fiscal instruments offer value-adding contributions to the beef, seafood and poultry business. According to Tenrisanna and Kasim [14], providing appropriate subsidies to livestock producers by the government is an instrument that needs to be applied to achieve a self-sufficiency program that facilitates increased livestock production. Subsidies provided to livestock farmers can help cut production costs, which might lower the domestic prices of meat such as beef. Household animal-sourced protein demand elasticities provided by Anindita et al. [56] and Khoiriyah et al. [45] suggest that a change in the domestic prices of meats such as beef, poultry, and seafood is expected to bring about a more than proportionate change in the quantity demanded. Hence, subsidies to livestock producers can help to increase domestic supply, which can then help to reduce prices and foster higher levels of domestic consumption. Furthermore, Forgenie and Khoiriyah [95] found that domestic production and food imports share an inversed relationship, in that increased domestic production of food leads to a more than proportionate decrease in food imports. Therefore, investments in constructing modern abattoirs and slaughterhouses in production centers can provide the infrastructure needed to help increase domestic meat output and improve animal-derived protein self-sufficiency in Indonesia. On the other hand, since subsidy is an expenditure and a cost to the government, it may be more value-adding if the Indonesian government uses the funds to invest in infrastructural development of the meat and dairy sector. This may have a more direct impact on producers, consumers and those involved in the value-chain.

Applying a tax on imports can be a valid policy option to reduce excessive imports by nations [96,97]. If domestic production can meet domestic demand, the government and policymakers can consider taxing specific imported animal-sourced proteins such as imported poultry and seafood. Implementing a tax on these items would cause them to be relatively more expensive than locally produced items, assuming that domestic prices are lower than import prices due to the implementation of the tax. Since imported poultry and seafood were found to have inelastic demand, applying a 10% import tax on these items would lead to an 8.65% and 8.79% decrease in import quantity, respectively. The income generated from the tax can then be used to aid in financing programs and training to help boost domestic production. Governments can also consider developing and implementing strict food safety regulations to help control excessive imports [98]. This might help reduce the volume of imported meats and seafood. However, to avoid negative feedback, such tax policies would only be effective and beneficial if Indonesia increased its domestic production. To put this into proper context, imposition of an import tax may have a blow-back effect on the Indonesian people if domestic production falls short of demand and there is heavy reliance on importation of meat and dairy products. This will have a spillover and multiplier effect of increasing production cost and overall price level in the meat, dairy, and seafood sector.

## 4 Conclusion

This study utilized both the static and an error-corrected version of the LAAIDS model to study imported meat and seafood demand in Indonesia over the period 1976–2020 using annual data. Two tests for cointegration were used in the Engle-Granger two-step residual-based and dynamic approaches. Both tests suggested a long-run equilibrium relationship among the variables in the system of equations, and a dynamic specification fits the data. This was further confirmed by the theoretical restrictions of homogeneity and symmetry, being rejected in the long-run model but held in the short-run model. It was discovered that habit formation behavior significantly determines budget share expenditure for imported seafood, pork, and poultry in the short-run but does not affect the demand for imported beef and mutton. In addition, it was found that imported mutton had the slowest reversion to long-run equilibrium compared to other imported items, which had a moderate reversion speed.

The study also calculated income and price elasticities of demand for imported meats and seafood for both time horizons. Income elasticities reveal that imported beef was the most responsive to changes in income in the short-run and was a luxury good but was a normal good in the long-run. Imported poultry was found to be the least responsive to changes in income in the short-run, but imported seafood was the most responsive to income changes in the long-run. In the long-run, all imported meats were found to be luxuries except for imported seafood and beef. Own-price elasticities of demand reveals that in the short-run all imported items had inelastic demand except for imported beef which had elastic demand. In the long-run, however, imported beef and pork had elastic demand, while all other items were inelastic. The study also found that substitution relationships existed among pairs of imported commodities. Finally, a few policy suggestions were discussed, such as improved infrastructure for domestic production and production subsidies to producers.

While our study on import demand for meat and seafood in Indonesia from 1976 to 2020 offers crucial insights, it has certain limitations that should be acknowledged. Firstly, the use of aggregated annual data might obscure short-term dynamics, warranting future investigations with more granular data. Secondly, the annual frequency of the data used could potentially mask intra-year fluctuations and variations in consumer behavior, leading to a less nuanced understanding of import demand patterns. Maybe quarterly or monthly data should be considered, although such data is usually unavailable. Finally, the reliance on unit price as a proxy for import prices introduces potential inaccuracies. Nevertheless, our study remains a valuable contribution by providing vital policy-relevant information on import price and income elasticities, filling a critical gap in the literature. Despite these limitations, our research findings underscores the significance of understanding consumer behavior and informs policy decisions, shedding light on strategies for import demand management and production subsidies in Indonesia's agricultural and trade sectors.

## Data availability statement

Data will be made available on request by contacting the corresponding author.

## Ethical statement

The authors would like to inform you that no humans or animals were involved in this paper.

## Funding statement

The authors would like to inform you that this research did not receive any specific grant from funding agencies in the public, commercial, or not-for-profit sector.

## CRediT authorship contribution statement

**David Forgenie:** Conceptualization, Data curation, Formal analysis, Methodology, Software, Supervision, Validation, Writing – original draft, Writing – review & editing. **Nikmatul Khoiriyah:** Conceptualization, Methodology, Resources, Validation, Writing – original draft, Writing – review & editing. **Meera Mahase-Forgenie:** Data curation, Methodology, Resources, Visualization, Writing – original draft, Writing – review & editing. **Bosede Ngozi Adeleye:** Resources, Validation, Writing – original draft, Writing – review & editing.

## Declaration of competing interest

The authors declare that they have no known competing financial interests or personal relationships that could have appeared to influence the work reported in this paper.

## Figures and Tables

**Table A1:** Phillips-Perron Test for Unit Roots

Series	Level Series		First Difference Series	
	No Trend	Trend	No Trend	Trend
Budget Shares				
S_1_	−2.870	−3.387	−7.902	−7.842
S_2_	3.656	−4.492	−7.684	−7.601
S_3_	−2.565	−4.797	−8.573	−8.469
S_4_	−1.401	−4.141	−8.111	−8.097
S_5_	−3.241	−3.668	−6.370	−6.296
Log Price and Expenditure				
lnp_1_	−1.961	−3.151	−6.537	−6.475
lnp_2_	−1.478	−2.784	−8.691	−8.602
lnp_3_	−2.709	−3.408	−8.091	−7.985
lnp_4_	−1.461	−1.528	−6.127	−6.124
lnp_5_	−1.565	−1.883	−7.825	−7.829
ln (M/P)	−1.31	−4.667	−7.418	−7.358
Critical Values				
1%	−3.589	−4.181	−3.592	−4.186
5%	−2.929	−3.516	−2.931	−3.518
10%	−2.603	−30188	−2.604	−3.189

Note: ln = natural logarithm, S = budget share and p = price, 1 = seafood, 2 = beef, 3 = pork, 4 = poultry, and 5 = mutton.

**Table A2:** KPSS Test for Stationarity

Series	Level Series		First Difference Series	
	No Trend	Trend	No Trend	Trend
Budget Shares				
S_1_	0.888	0.220	0.053	0.043
S_2_	0.819	0.127	0.040	0.030
S_3_	1.601	0.085	0.022	0.022
S_4_	1.755	0.186	0.074	0.022
S_5_	0.667	0.119	0.036	0.031
Log Price and Expenditure				
lnp_1_	1.763	0.090	0.046	0.030
lnp_2_	1.651	0.297	0.054	0.048
lnp_3_	1.243	0.264	0.084	0.085
lnp_4_	0.427	0.373	0.114	0.089
lnp_5_	1.054	0.389	0.086	0.085
ln (M/P)	2.221	0.111	0.032	0.019
Critical Values				
1 %	0.739	0.216	0.739	0.216
5 %	0.463	0.146	0.463	0.146
10 %	0.347	0.119	0.374	0.119

Note: ln = natural logarithm, S = budget share and p = price, 1 = seafood, 2 = beef, 3 = pork, 4 = poultry, and 5 = mutton.
